# An initial validation of Landsat 5 and 7 derived surface water temperature for U.S. lakes, reservoirs, and estuaries

**DOI:** 10.1080/01431161.2018.1471545

**Published:** 2018-05-10

**Authors:** Blake A. Schaeffer, John Iiames, John Dwyer, Erin Urquhart, Wilson Salls, Jennifer Rover, Bridget Seegers

**Affiliations:** a Office of Research and Development, U.S. Environmental Protection Agency, Research Triangle Park, NC, USA; b U.S. Geological Survey, Center for Earth Resources Observation and Science, Garretson, SD, USA; c Office of Research and Development, Oak Ridge Institute for Science and Education, U.S. Environmental Protection Agency, Research Triangle Park, NC, USA; d Goddard Space Flight Center, National Aeronautics Space Administration, Greenbelt, MD, USA; e Goddard Earth Sciences Technology and Research, Universities Space Research Association, Columbia, MD, USA

## Abstract

The United States Harmful Algal Bloom and Hypoxia Research Control Act of 2014 identified the need for forecasting and monitoring harmful algal blooms (HAB) in lakes, reservoirs, and estuaries across the nation. Temperature is a driver in HAB forecasting models that affects both HAB growth rates and toxin production. Therefore, temperature data derived from the U.S. Geological Survey Landsat 5 Thematic Mapper and Landsat 7 Enhanced Thematic Mapper Plus thermal band products were validated across 35 lakes and reservoirs, and 24 estuaries. *In situ* data from the Water Quality Portal (WQP) were used for validation. The WQP serves data collected by state, federal, and tribal groups. Discrete *in situ* temperature data included measurements at 11,910 U.S. lakes and reservoirs from 1980 through 2015. Landsat temperature measurements could include 170,240 lakes and reservoirs once an operational product is achieved. The Landsat-derived temperature mean absolute error was 1.34°C in lake pixels >180 m from land, 4.89°C at the land-water boundary, and 1.11°C in estuaries based on comparison against discrete surface *in situ* measurements. This is the first study to quantify Landsat resolvable U.S. lakes and reservoirs, and large-scale validation of an operational satellite provisional temperature climate data record algorithm. Due to the high performance of open water pixels, Landsat satellite data may supplement traditional *in situ* sampling by providing data for most U.S. lakes, reservoirs, and estuaries over consistent seasonal intervals (even with cloud cover) for an extended period of record of more than 35 years.

## 1. Introduction

Water quality is a critical consideration in determining water resource availability for human consumption, aquatic life, and recreation (U.S. EPA 2013). Temperature is relevant in nearly all biological and chemical processes, where the temperature coefficient (*Q*_10_) is defined as increased process rates with every 10°C temperature increase (Lighton 2008). The physical structure of the water column also is influenced by the creation of thermoclines and changes in the Brunt–Väisälä frequency as a measure of water column stratification, where the greater the frequency the greater the stratification (Schaeffer et al. 2012). Spatial and temporal measurements of temperature are key variables in ecological forecasting models and source water tracking. In particular, phytoplankton productivity (Eppley 1972), harmful algal bloom (HAB) toxin production (Davis et al. 2009), and forecasting (Wynne et al. 2013) may be parameterized with temperature. The United States Harmful Algal Bloom and Hypoxia Research Control Act (U.S. HABHRCA) of 2014 identified the need for forecasting and monitoring HABs in lakes, reservoirs, and estuaries across the nation. The ability to provide synoptic spatial and temporal satellite measurements of temperature may improve forecast modelling efforts for HABs, although these measurements offer a broad range of applications for other environmental monitoring purposes such as detecting thermal fronts and circulation patterns (Schwab et al. 1992).

Several satellite sensors are capable of collecting thermal emission data, but most do so at relatively coarse spatial resolutions. These satellites are effective at collecting sea surface temperature data, where spatial variation is low. Examples of ocean-based sensors with thermal detectors include the Advanced Very High Resolution Radiometer (McClain et al. 1985; Kilpatrick et al. 2001), Moderate Resolution Imaging Spectroradiometer (Minnett et al. 2002), and Visible Infrared Imaging Radiometer Suite (Petrenko et al. 2014). However, few thermal remote sensing options are available at the fine scale required for estimating surface temperatures of inland waters such as lakes and estuaries. Landsat and the Advanced Spaceborne Thermal Emission and Reflection Radiometer (ASTER) (Hulley et al. 2015; Abrams 2000) are the only publicly available satellite platforms that offer relatively fine spatial resolution thermal data, with resolutions ranging from 120 to 30 m.

Landsat resolution provides the potential to resolve >100,000 lakes and reservoirs in the contiguous U.S. (CONUS); however, validation efforts of satellite derived water quality products have been limited. Several previous studies have validated the Landsat thermal bands, though most studies investigated either single or a select few waterbodies. Larger waterbodies studied include the Michigan and Ontario Great Lakes, Lake Tahoe, Lake Constance in Germany, and Chesapeake Bay. Lathrop and Lillesand (1987) used measurements from 15 locations in Lake Michigan to build an algorithm for temperature from Landsat 5. Calibration of the Landsat 7 thermal bands using *in situ* measurements from nine collection sites was demonstrated in Lake Ontario (Schott et al. 2001). Barsi et al. (2003) used several *in situ* temperature datasets from Lake Tahoe to calibrate Landsat 5 and Landsat 7. In a related study, Hook et al. (2004) compared Landsat radiance from 24 images to radiance predicted based on *in situ* temperature measurements at four points in Lake Tahoe. At Lake Constance, Germany, Schneider and Mauser (1996) validated temperature estimates from 21 Landsat images to sonde data taken within 60 min of satellite overpass. Landsat-derived temperature was validated using 196 images with daily *in situ* temperature data from one location in the Chesapeake Bay (Ding et al. 2015). The Embalse del Rio Tercero reservoir was used as a case study by Lamaro et al. (2013) to validate temperature estimates from five Landsat images with *in situ* temperature data from seven locations in Argentina. Some studies investigated multiple waterbodies but over limited spatial extents. Wukelic et al. (1989) investigated the accuracy of Landsat temperature at eight sites along the Columbia and Yakima Rivers; and Simon et al. (2014) studied two lakes in France, comparing 145 Landsat images to *in situ* measurements from one location in each lake.

Though substantial effort has been put forth to validate the thermal band performance of various satellites over the past several decades, there is a paucity of research that does so both at a fine spatial resolution and across a broad spatial extent relevant for management applications. Until such a broad validation is performed, reliable remote sensing of waterbody surface temperature remains restricted to individual waterbodies with available *in situ* data. On the other hand, the wide usage of Landsat temperature algorithms enabled by broader validation efforts would make waterbody temperature data more available to municipalities and other stakeholders lacking the resources to perform field measurements.

This study leverages spatially extensive lake, reservoir, and estuarine temperature datasets to provide a broad-scale validation of Landsat surface temperature data for lakes across CONUS and a variety of ecoregions. The objectives of this study are to (1) determine the number of surface lake and reservoir waterbodies sampled for temperature through traditional *in situ* stations compared to Landsat and (2) validate the water application of a U.S. Geological Survey (USGS) land surface temperature provisional product in ecologically diverse lakes, reservoirs, and estuaries.

## 2. Methods

This section discusses the study methods, including national *in situ* water temperature measurements, Landsat temperature data, Landsat validation with *in situ* data, and *in situ* locations for data validation.

### 2.1. National in situ water temperature measurements

*In situ* discrete measurements of temperature were accessed through the Water Quality Portal (WQP, www.waterqualitydata.us). The WQP website serves data collected by state, federal, and tribal groups across the United States from the USGS National Water Information System (NWIS), U.S. Environmental Protection Agency (U.S. EPA) Storage and Retrieval Data Warehouse (STORET), and U.S. Department of Agriculture (USDA) Sustaining The Earth’s Watersheds Agricultural Research Database System (STEWARDS). Data were selected from WQP based on the following criteria: estuary, lake, reservoir, or impoundment site type; water sample medium; physical characteristic group; and water temperature characteristic. The data were for the period 1 January 1980 through 1 January 2016 within the continental United States (CONUS). Discrete *in situ* measures were retained for temperature (°C) Landsat validation if they were in the upper 0.5 m of the water column. Only USGS NWIS *in situ* temperature measurements were used for Landsat temperature validation to maintain a consistently high-quality (Kilpatrick et al. 2001) set of spatial and temporal match-up observations.

### 2.2. Resolvable lakes and reservoirs

Resolvable lakes and reservoirs were previously calculated from Clark et al. (2017) for those that fit the U.S. EPA National Lakes Assessment (NLA) 2012 site evaluation criteria using the method briefly summarized here. A waterbody mask was generated using the National Hydrography Dataset (NHD) Plus version 2.0 (NHDPlusV2, https://viewer.nationalmap.gov ) (McKay et al. 2012) to identify waterbodies resolved with 30 m pixel resolution, assuming a minimum three-by-three (3 × 3) pixel array requirement. The waterbody spatial coverage with resolvable satellite pixels was calculated based on the minimum Euclidian distance from shore that will accommodate the 3 × 3-pixel or larger array. The Landsat surface temperature data were interpolated to 30 m to ensure alignment with the solar reflective bands in the Level-1 products, which were in turn used as inputs to the surface temperature retrieval algorithms.

### 2.3. Landsat temperature data

Landsat surface water temperature (SWT) was derived from the USGS land surface temperature provisional product (Cook et al. 2014; Hulley et al. 2014; Hulley et al. 2009; Hulley et al. 2015; Laraby et al. 2016; Hook et al. 2004). The land surface temperature provisional products were derived using Landsat 5 Thematic Mapper (TM) and Landsat 7 Enhanced Thematic Mapper Plus (ETM+) thermal data, North American Regional Reanalysis (NARR) weather data, the Moderate Resolution Atmospheric Transmission (MODTRAN) model, and ASTER emissivity data (Cook et al. 2014). Previous efforts report that Landsat data under ideal atmospheric conditions underestimated the surface temperature by 0.26 K, whereas an unstable atmosphere with cloudy conditions could result in errors as large as several degrees (Laraby et al. 2016). Therefore, to avoid large errors in the surface temperature, only Landsat scenes with <10% cloud contamination were selected for this study. For this validation effort, relatively cloud-free Landsat scenes within ±3 days of coincident *in situ* measures were retained for validation across the CONUS.

### 2.4. Landsat validation with in situ data

Lake and reservoir shoreline polygons were extracted from the NHDPlusV2. For estuarine shoreline, the National Oceanic and Atmospheric Administration (NOAA) Medium Resolution Shoreline (https://shoreline.noaa.gov/data/datasheets/medres.html) was selected for estuarine temperature validation based on exceedance of National Map Accuracy Standards and an average scale of 1:70,000. For lake and reservoir validation, two sets of temperature data were evaluated: land-water boundary (<59 m from shore) data and far shore (>180 m from shore) data. Estuary data were retained and evaluated only if they were >180 m from shore. The distance of >180 m from shore was selected to avoid pixels contaminated by a land signal for both TM and ETM+. The diagonal distance of a 120 m Landsat 5 TM pixel was calculated with the equation *D* = *R*×√2, where *D* is the diagonal distance and *R* is the length of the pixel side (120 m). The distance was increased to >180 m, to account for the Landsat geolocation uncertainty of <12 m (Irons et al. 2012). This ensured an *in situ* sampling point that occurred at the edge of the diagonal distance was not matched to a mixed pixel and lake points were centred 100% over water, with no land contamination. The 59 m near shore threshold was chosen to be smaller than the finer of the two thermal band spatial resolutions for both the Landsat 7 ETM+ (60 m) and Landsat 5 TM (120 m) sensors. This <59 m near shore threshold ensured that any near-shore point had the probability of falling (1) within a 100% water 30 m SWT pixel adjacent to a ‘mixed’ (land and water) SWT pixel or (2) within a ‘mixed’ (land and water) 30 m SWT pixel. Statistics generated included the bias and mean absolute error (MAE). The results were presented in a star plot as a convenient way to summarize and compare performance (Seegers et al. 2018).

### 2.5. In situ locations for data validation

*In situ* observations for data validation were selected across a diverse geographic area under a variety of atmospheric and aquatic conditions (Kilpatrick et al. 2001) to ensure a comprehensive validation dataset. Representative estuaries were selected from the NOAA National Estuarine Eutrophication Assessment regions (Table 1; Bricker et al. 2007). Mobile Bay is an open bay system in the Gulf of Mexico with a subtropical climate and annual air temperatures of 22°C. Mid-Atlantic estuaries include New York and New Jersey locations and coastal ocean sites with temperate climates and annual air temperatures of 12°C. South Carolina estuaries are in the South Atlantic region in a temperate climate with annual mean air temperatures of 19°C. Puget Sound is a fjord system in the Pacific region with annual air temperature of 10°C. Barnegat Bay, Mobile Bay, and Puget Sound estuaries also are identified as part of the National Estuarine Program to restore water quality and ecological integrity.

Representative lakes were selected from the nine National Aquatic Resources Survey ecoregions (Herlihy et al. 2008). The lakes by region are summarized in Table 1. The Coastal Plains region includes the Gulf Coast from Florida to eastern Texas with flat topography. Lake Champlain is a major waterbody in the Northern Appalachians region that includes the Adirondack Mountains within the cold climate zone. The Northern Plains region is dry, with short summers and long winters. The Southern Appalachians region has a wet temperate climate contrary to the Southern Plains region, which has a dry temperate climate. The Temperate Plains region is mild in climate, with balanced winters and summers. The Upper Midwest region is known for cold winters and short summers. The Western Mountains region is sub-arid and the Xeric region is warm and dry.

## 3. Results and discussion

### 3.1. National temperature measures

**Figure 1.**
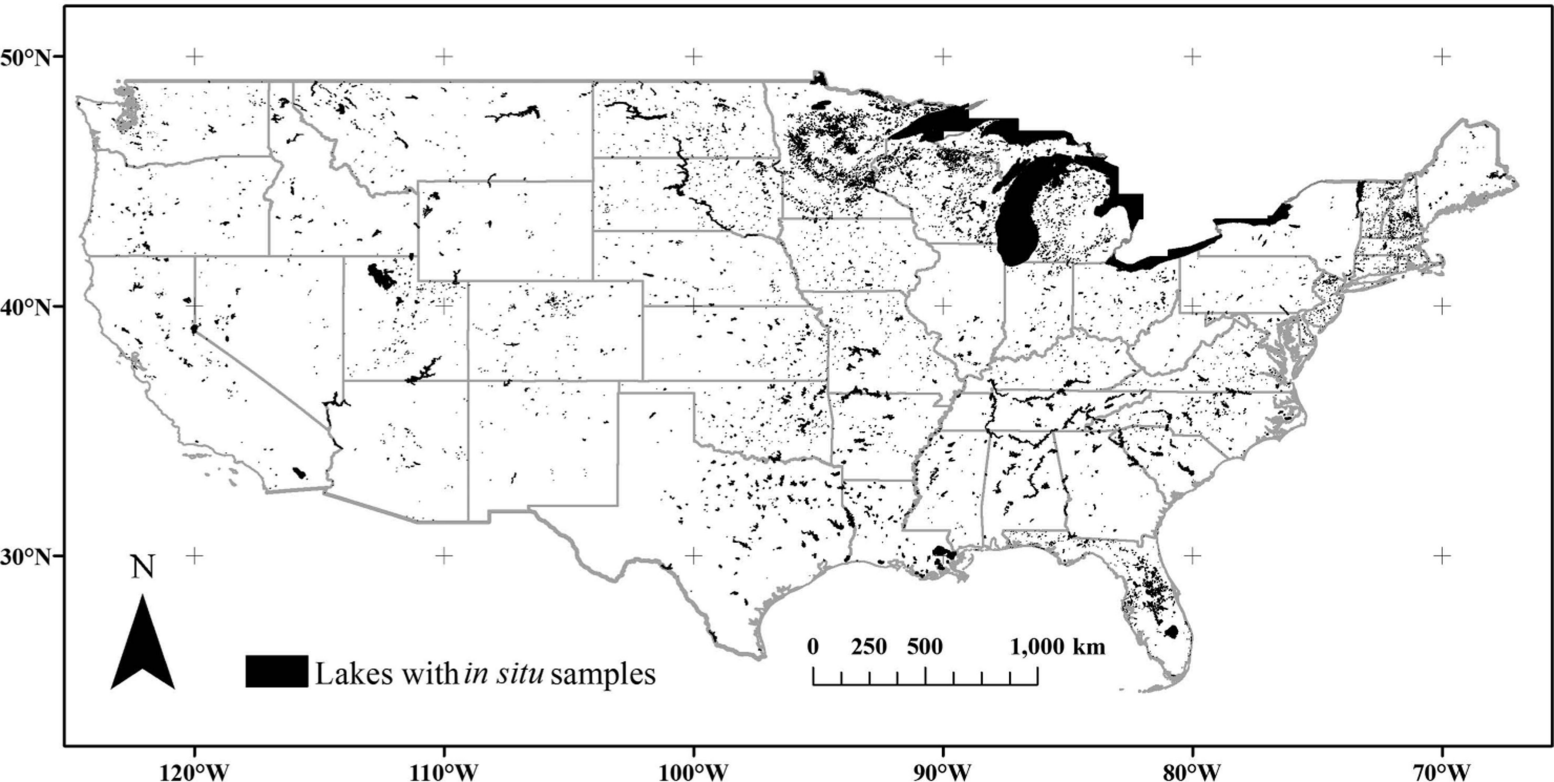
Locations of 11,910 lakes and reservoirs with discrete *in situ* sampling stations from the WQP from 1980 through 2015. Of the 275,897 U.S. lakes and reservoirs, 95.68% have no WQP temperature measurements.

The WQP returned 38,724 station locations, 30,254 from STORET and 8,470 from NWIS including 3,746,474 discrete *in situ* temperature samples. A total of 11,910 out of 275,897 (4.3%) NLA 2012 NHDPlusV2 waterbodies were sampled for temperature within a 100 m buffer from 1980 through 2015 (Figure 1) and 95.7% had no *in situ* temperature measurements from the WQP. All CONUS states had *in situ* temperature measurements. The lowest number of sampled lakes and reservoirs were in the District of Columbia with three waterbodies and in West Virginia with 13 waterbodies. The greatest number of lakes sampled for *in situ* temperature were in Florida with 6238 and Minnesota with 5961 waterbodies. Water quality data, including temperature measurements, often are collected by a variety of local government and non-government organizations. Unfortunately, the WQP does not have representation from all these individual organizations, because these data sources may be difficult to obtain and inconsistent in the way the data are disseminated. Therefore, WQP results under-represent total sampling from all groups. Although lacking data from these other groups the WQP does provide an opportunity to openly access standardized water quality data records through a simple web service (Read et al. 2017). Most CONUS waterbodies continue without sampling based on WQP analysis, because of resource constraints, time investment, difficult terrain, and the sheer number of lakes. Waterbodies in the WQP have sampling coverage from sparse to heavy, depending on programmatic support and employee level of effort.

**Figure 2.**
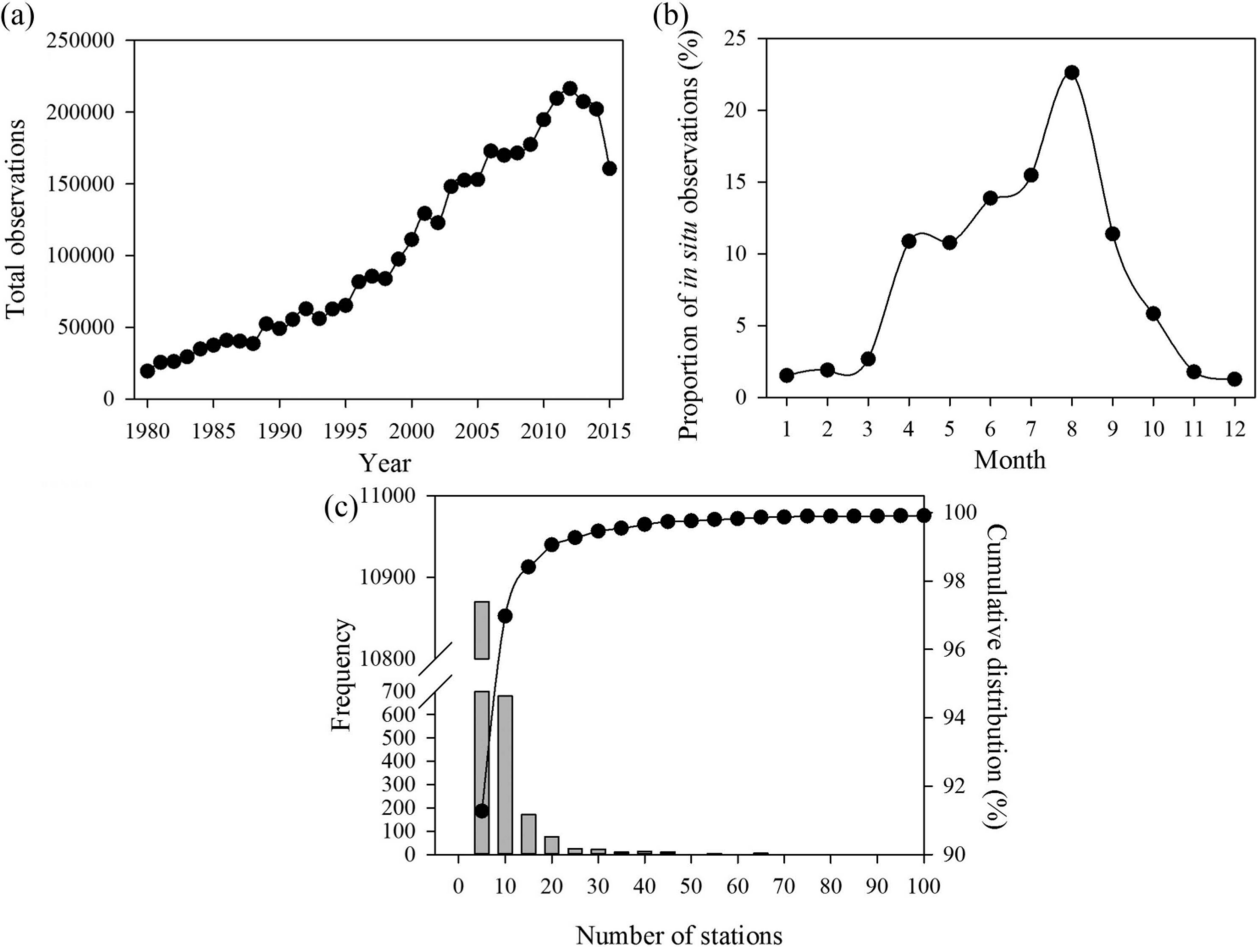
Number of *in situ* station observations by (a) year, (b) percent of total samples by month, and (c) number of stations per lake and reservoir (bars) and cumulative distribution of stations per lake and reservoir (points).

Total *in situ* temperature sampling events increased 1013% from 1980 through 2012 (Figure 2a) with a recent −4.2% year-to-year decline in 2013, another −2.5% year-to-year decline in 2014 and a −20.5% year-to-year decline in 2015. Though the total number of observations has substantially increased over the 1980s, this trend was broken, especially during the most recent years from 2012 through 2015. The recent decline in observations may be due to reduced resources or a lag in uploading data to online databases such as STORET.

Sampling rates vary seasonally throughout the year. The greatest number of *in situ* observations (85.0%) occurred from April through September, with the remaining 15.0% occurring from October through March (Figure 2b). Peak sampling occurred in August, with 22.6% of the observations. The least number of samples occurred in December (1.3%). Seasonal sampling was biased heavily towards warmer months, with underrepresentation from November through March, typically the coldest months of the year. Most waterbodies (91.3%) contained ≤5 stations, and the majority (55.6%) only have a single sampling station (Figure 2c). Landsat data can supplement *in situ* data, especially during the colder months, with long-term seasonal measurements and provide per-pixel measurements for temporal and spatial waterbody coverage.

**Figure 3.**
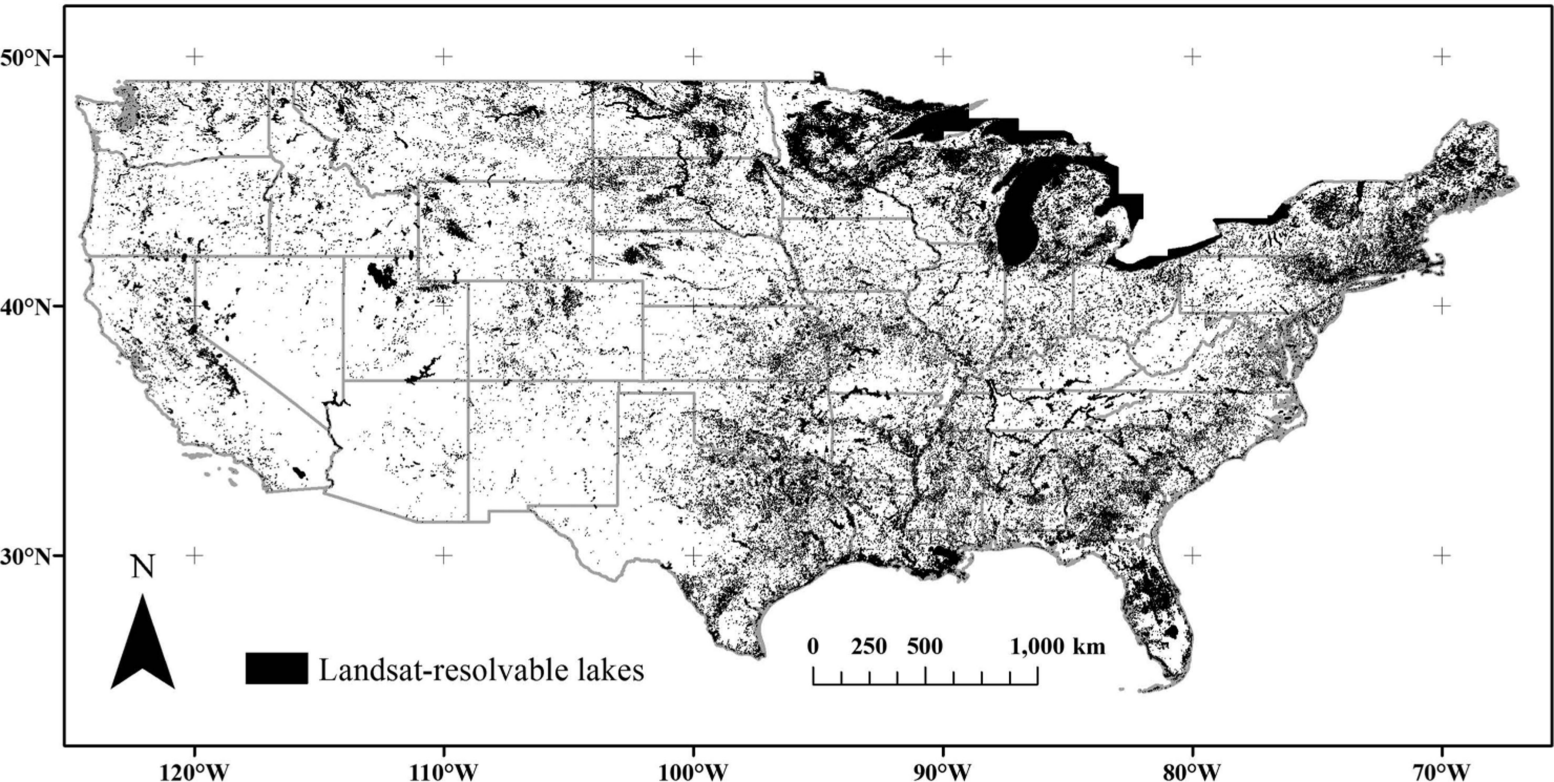
Locations of 170,240 lakes and reservoirs resolved by Landsat, where 38.29% of the 275,897 U.S. lakes had no temperature measurements.

**Figure 4.**
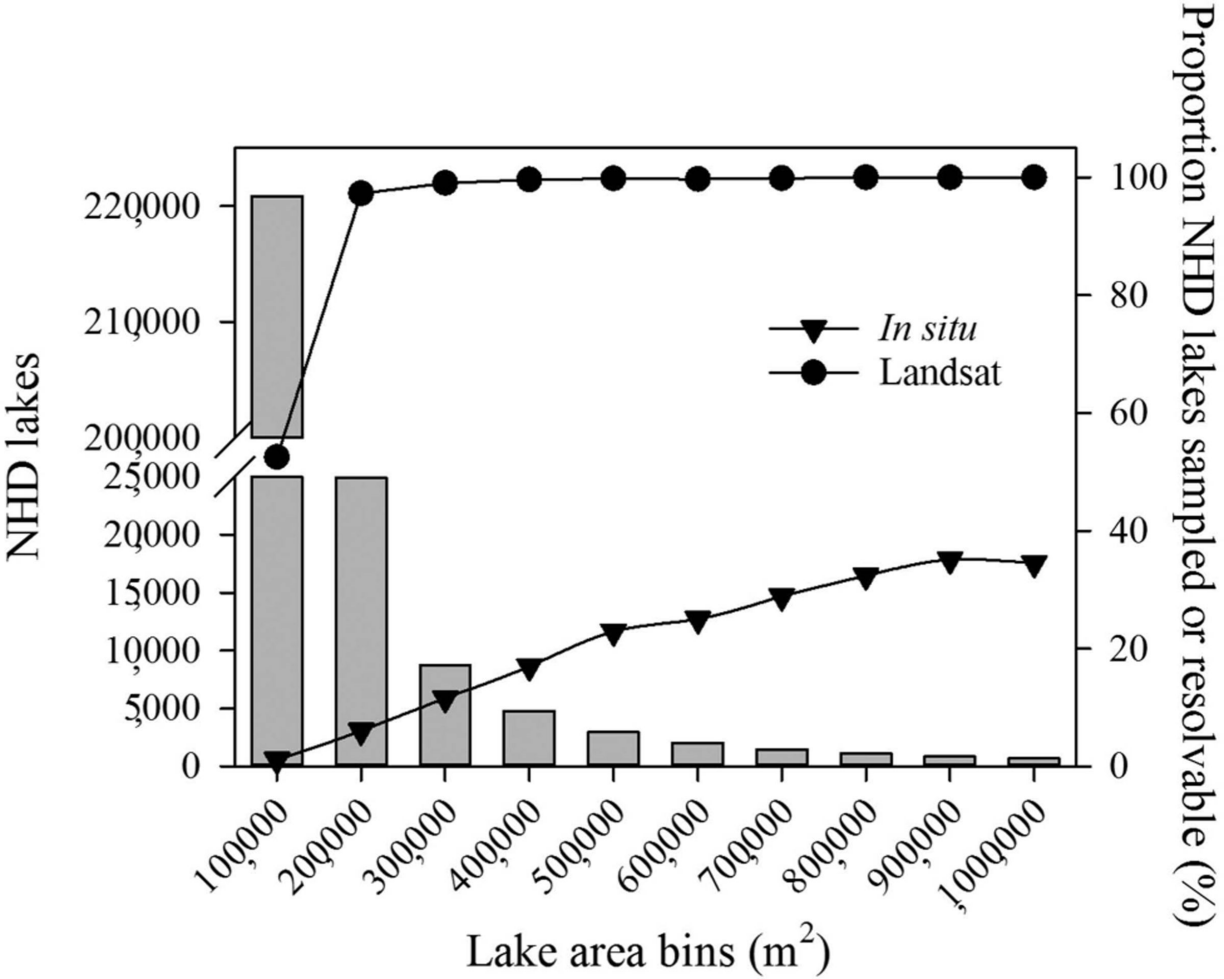
Number of NHDPlusV2 lakes and reservoirs (bars), percentage of NHDPlusV2 lakes and reservoirs with *in situ* samples (triangles), and percentage of NHDPlusV2 lakes and reservoirs resolvable with Landsat (points) in each surface area category.

Of the 275,897 lake and reservoir NHDPlusV2 waterbodies that met the U.S. EPA NLA 2012 criteria, 170,240 (62%) were resolvable by a window width 90 m wide, a resolution equivalent to a 3 × 3-pixel array (Figure 3). Most (80%) of the waterbodies were ≤100,000 square metres (m^2^), and Landsat could provide measurements in 52.5% of these lakes, which is a large improvement compared to the 1.1% of the lakes and reservoirs with *in situ* sampling (Figure 4). The monitoring of smaller lakes and reservoirs is important because the total worldwide contributions of these waterbodies have influence on global scale system dynamics (Downing et al. 2006). Both *in situ* sampling and Landsat coverage improved as waterbody size increased. Landsat provided 100% coverage for waterbodies measuring ≥800,000 m^2^, whereas *in situ* sampling was completed in 35% of these large lakes.

Landsat data can supplement traditional *in situ* sampling by providing data for most CONUS lakes and reservoirs (62%) over seasonal intervals, even with cloud cover, for at least 35 years. The 30 m Landsat dataset provides more measurements for smaller lakes and reservoirs than representation from traditional sampling. Programs that combine a variety of monitoring methods such as discrete *in situ* sites, synoptic measurements from satellites, continuous sensors, and models may improve management strategies for water quality problems (Pellerin et al. 2016).

### 3.2. Landsat SWT validation

**Figure 5.**
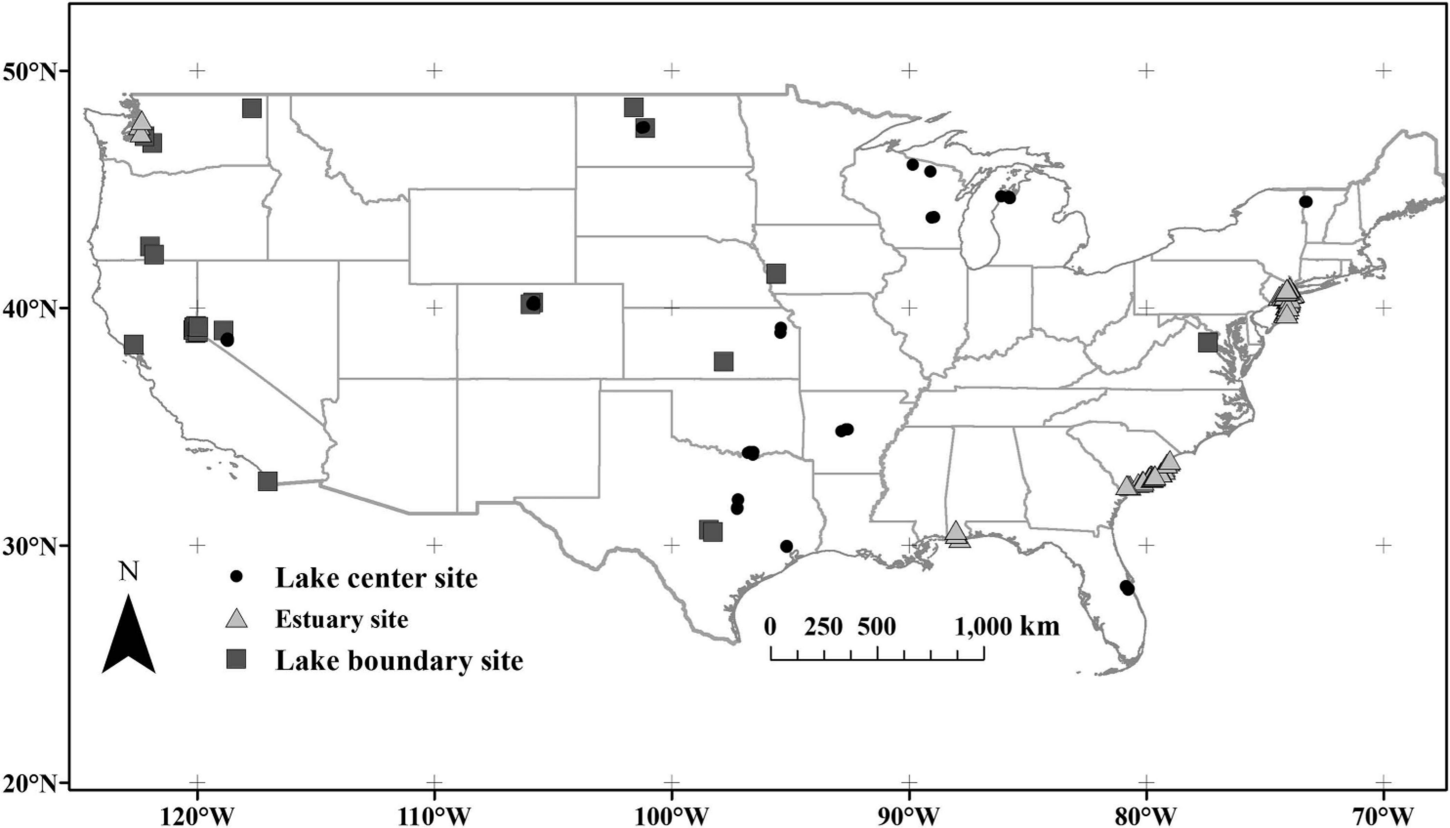
Locations of 35 lakes and 24 estuaries used for Landsat 5 and Landsat 7 SWT validation. *In situ* validation locations were selected >180 m from land (black circles), at the land-water boundary (dark grey squares), and in estuarine locations >180 m from land (grey triangles).

**Figure 6.**
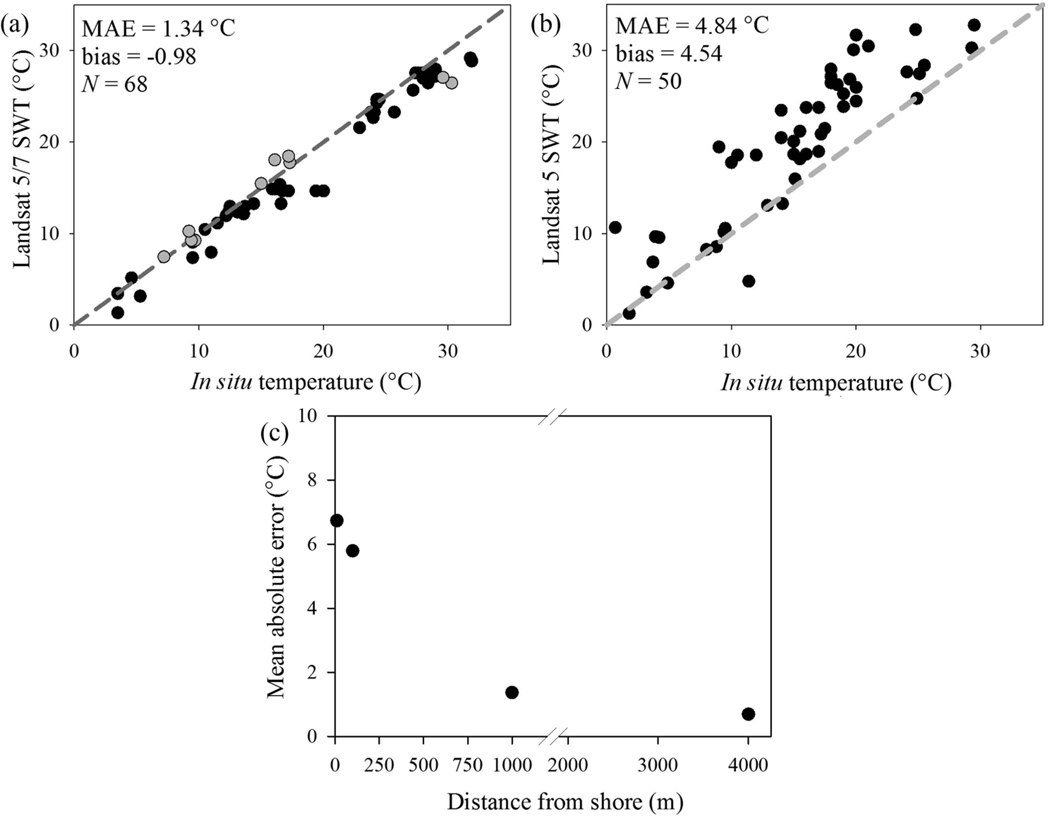
(a) Validation of Landsat 5 (black circles) and Landsat 7 (grey circles) SWT with *in situ* temperatures from the WQP for lake and reservoir locations the >180 m from land. *N* is the number of validation points. (b) Validation of Landsat 5 SWT with *in situ* temperature for lake and reservoir land-water boundary locations. (c) Change in MAE for both >180 m lake, reservoir, and land-water boundary locations binned from 0 to 10 m, 11 to 100 m, 101 to 1000 m, and 1001 to 4000 m from shore.

**Figure 7.**
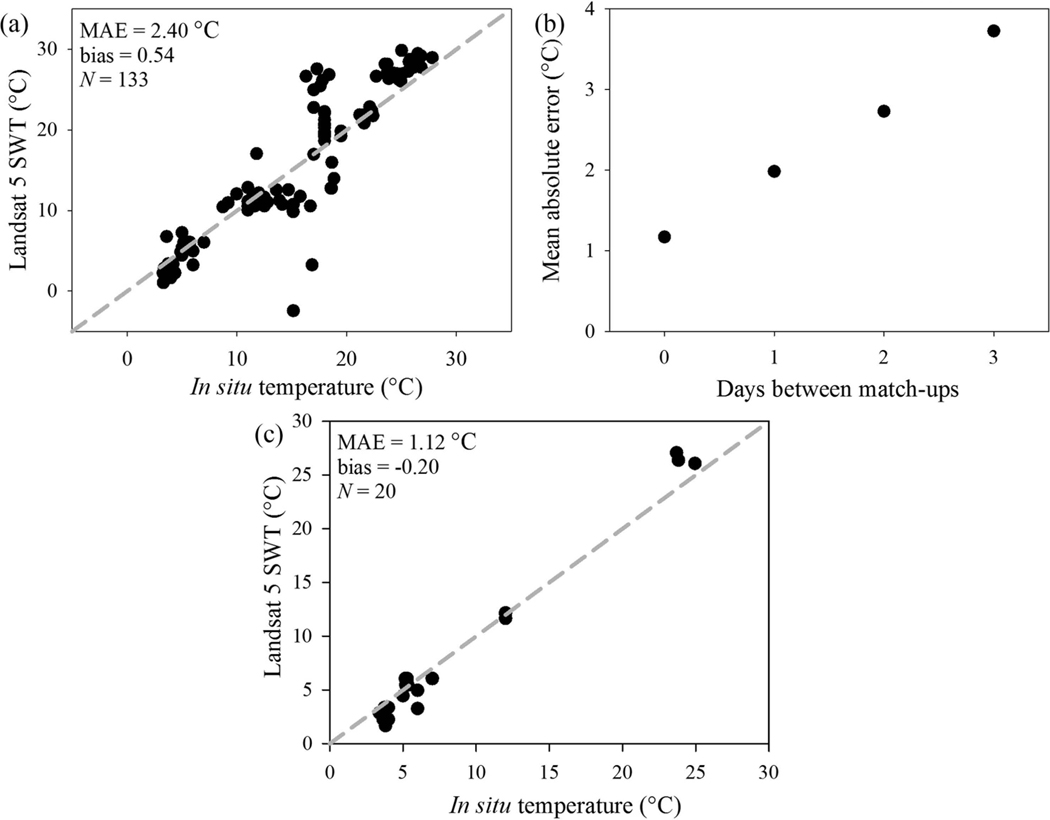
(a) Validation of Landsat 5 SWT with *in situ* temperature from the Water Quality Portal for the ±3 days and >180 m from land estuarine locations. *N* is the number of validation points. (b) Change in absolute error for estuary locations with number of days between match-ups. (c) Validation of Landsat 5 SWT in estuaries from only match-ups within the same day and >180 m from land.

Limitations in the number of match-ups included sample density after meeting defined filtering requirements (see sections 2.1, 2.3, and 2.4), image processing time, and operator time since match-ups were conducted by hand to assure avoidance of scene contamination such as clouds, smoke, boat wake, and scan line corrector failure in Landsat 7. The WQP returned 118 temperature match-ups across 35 lakes and reservoirs and 133 paired observations for 24 estuaries (Figure 5). SWT match-ups >180 m from land closely followed the 1:1 line, with MAE 1.34°C (Figure 6a). SWT match-ups at the land-water boundary were positively biased (4.54) above the 1:1 line and had increased mean error (MAE 4.84°C; Figure 6b). Average error increased with proximity to land with a high MAE of 6.73 ^o^C <10 m from the NHDPlusV2 shoreline (Figure 6c). Bias in the match-ups with proximity to land were attributed to the emissivity signal contribution from land in these mixed land-water pixels. Estuary SWT match-ups followed the 1:1 line, with MAE 2.40°C (Figure 7a). Average error increased with each subsequent day between the satellite overpass and *in situ* sample, where ±3-day match-ups had MAE 2.40°C (Figure 7b) compared to same day match-ups that had 1.12°C MAE (Figure 7c).

**Figure 8.**
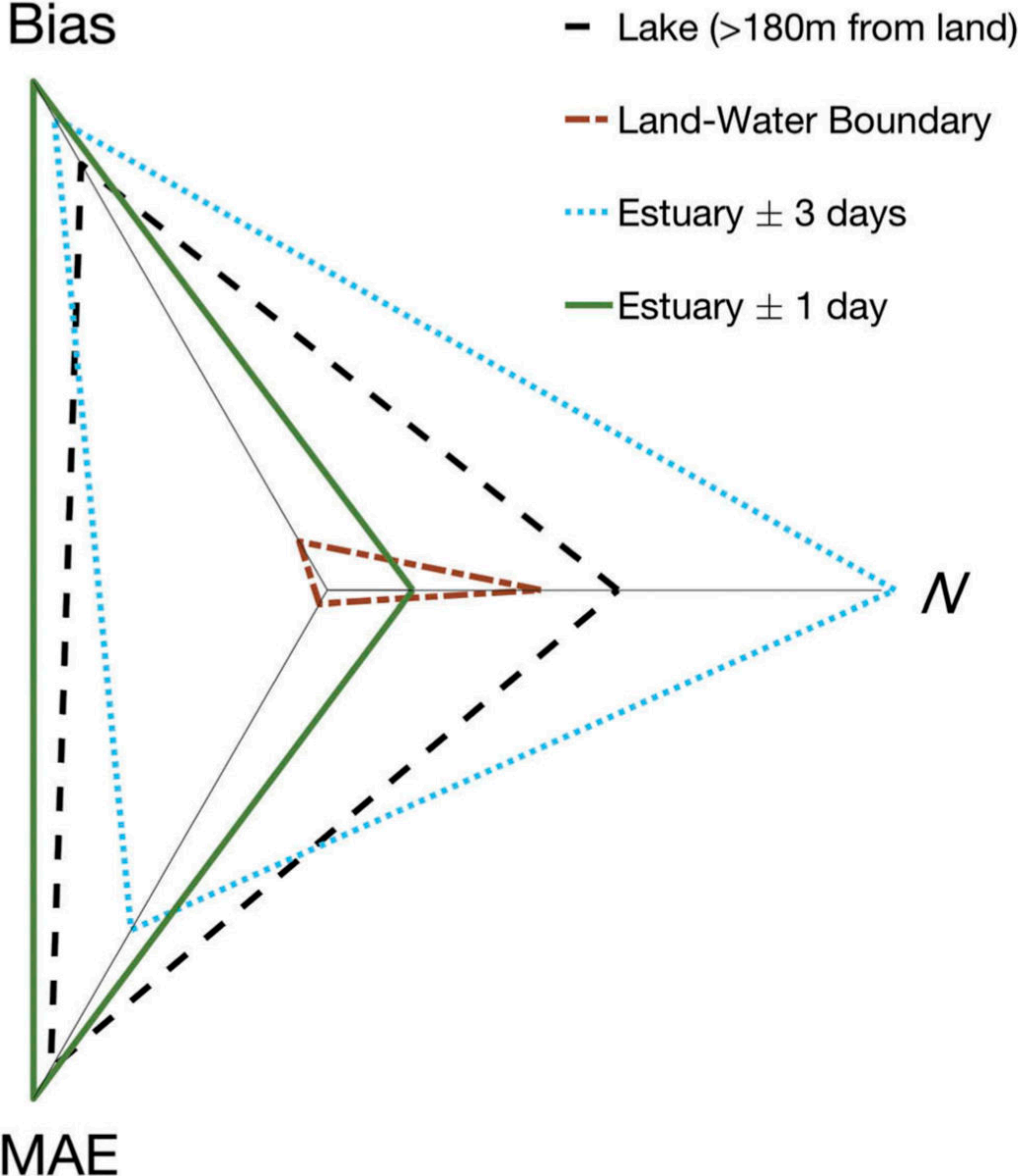
Star plot comparison of metric results of bias, MAE, and number of samples (*N*) summarized for locations >180 m from land, land-water boundary locations, estuary ±3 day, and estuary same day. The plot centre represents values with poorer algorithm performance, and the maximum length of each spoke reveals best algorithm performance.

**Figure 9.**
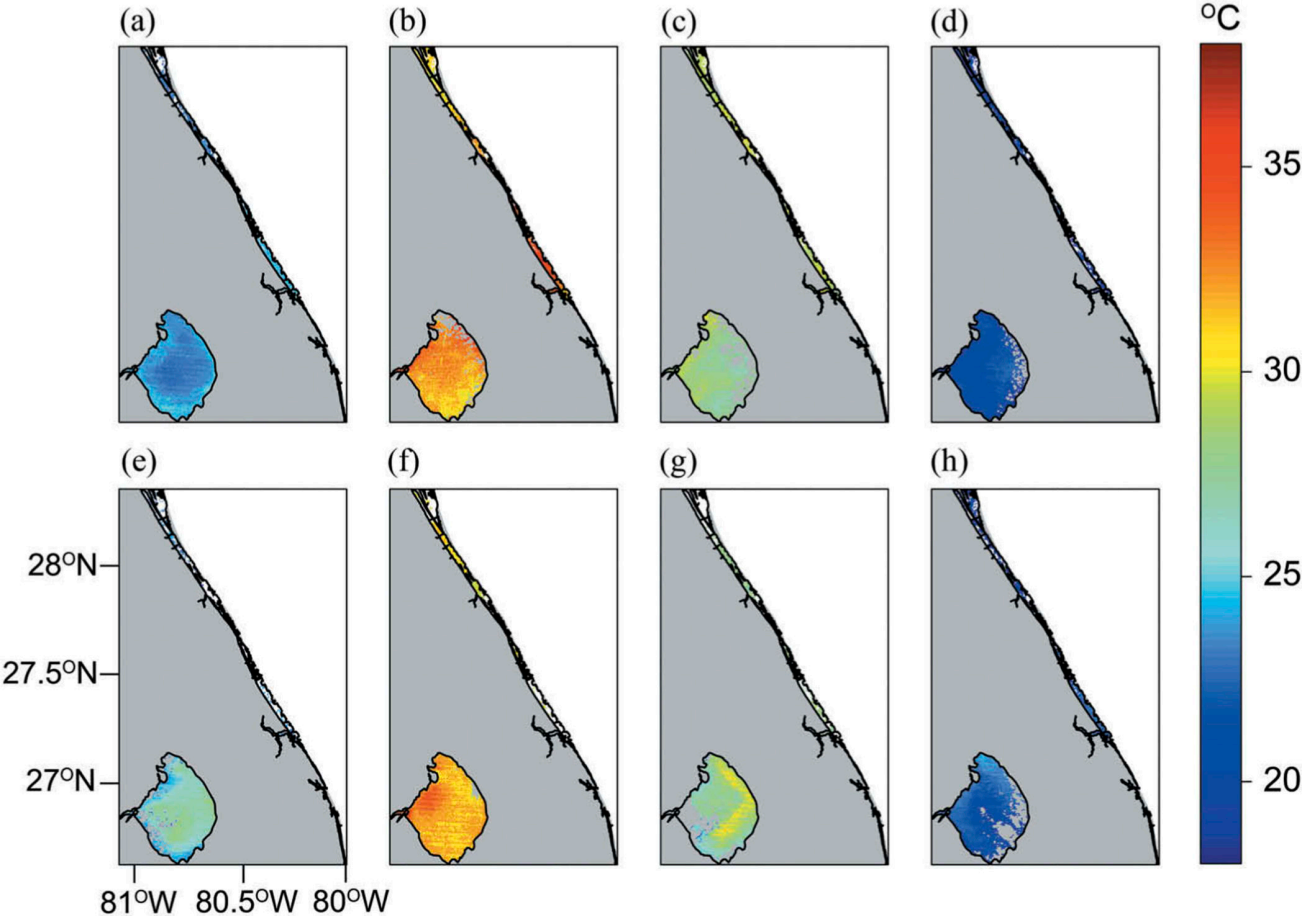
Representative Landsat 5 seasonal scenes of temperature in Indian River Bay and Lake Okeechobee, Florida. Winter 2008 are figure tiles a through d from spring, summer, fall, and figure tiles e through h are seasonal scenes from 2011. Grey colour represents land and clouds.

A summary of algorithm performance metrics were compared with a star plot for >180 m from land, land-water boundary sites, estuaries ±3 days, and estuaries same day results (Figure 8). Star plots provide an effective graphical approach to evaluate algorithm performance across multiple metrics (Chambers et al. 1983; Cisneros et al. 2014). Star plots visually consolidate our algorithm performance assessment across all recommended metrics (bias, MAE, and number of match-ups), thus providing a convenient tool for visually comparing results and differences. Finally, scenes from Landsat 5 were selected to demonstrate both spatial and temporal continuity in southeastern Florida for Indian River Bay and Lake Okeechobee (Figure 9). The spatial and temporal stability of the algorithm suggests that it could be sufficient for determining historical seasonal trends and assessing inter-annual variability of lake, reservoir, and estuarine temperatures.

Hook et al. (2004) reported bias from Landsat 5 between 0.2°C and 0.9°C, which is similar to the finding of this study of 0.98°C bias in lakes and reservoirs from Landsat 5 and 7 combined, and a bias of 0.2°C in Landsat 5 same-day estuary match-ups. Lamaro et al. (2013) reported standard errors of 1.04–1.22°C within a temperature range of 10–30°C using a ± 3-day match-up window. Wukelic et al. (1989) and Kay et al. (2005) also report Landsat SWT measures within 1–3°C of *in situ* validation data, which are similar to the result of 1.12–1.34° C MAE reported here within a 0–30°C range. Errors include spatial difference between single-point discrete samples, the heterogeneity of the water within a 30 m × 30 m integrated pixel sample, and time difference between the *in situ* sample and satellite overpass. This study used a commonly acceptable ±3-day match-up window to maximize the number of validation points (Lamaro et al. 2013; Simon et al. 2014; Bonansea et al. 2015). Lastly, Landsat SWT measurements were derived from the surface skin, which may produce results that vary from measurements taken at 0.5 m, depending on wind conditions at the site during sampling (Hook et al. 2004; Ding and Elmore 2015; Schneider et al. 1996).

The results of this study serve as an initial validation of the Landsat 5 and Landsat 7 satellite-derived SWT measurements across multiple waterbodies in all CONUS ecoregions. Landsat 5 and 7 provide records back to the 1980s that may benefit change detection methods for source tracking and HAB forecasting efforts. As expected, the SWT MAE results were higher at the land-water boundary than for pixels further from land. However, near-shore measurements will continue to be extremely important for protecting recreational use of waterbodies because humans may be exposed to surface water through dermal uptake during recreational swimming at the shin depth (0.3 m) or waist depth (1 m; Wade et al. 2010; Telecha et al. 2009).

## 4. Conclusion

This is the first study to quantify and identify Landsat resolvable lakes and reservoirs across CONUS, and compare traditional *in situ* sampling with an operational high-resolution satellite platform that will provide a long-term, consistently processed climate data record. To the authors knowledge, this is also one of the largest validation efforts of temperature for inland waters across a variety of ecosystems, other than Layden et al (2015) who validated 52 observations over 18 lakes for the Along-Track Scanning Radiometers. Although inland waterbody spatial and temporal representativeness was limited for *in situ* temperature samples, this study demonstrates the continued importance of these *in situ* datasets, which permitted the robust validation of the Landsat SWT algorithm and associated performance metrics in lakes, reservoirs, and estuaries across the CONUS. Comparison of *in situ* water temperature measurements with Landsat-derived SWT measurements indicates that absolute errors were within 2°C, and that the stability of the Landsat algorithm is sufficient for determining seasonal trends and assessing inter-annual variability of lake, reservoir, and estuarine temperatures. The satellite datasets provide a synoptic complement to discrete *in situ* sampling and may be considered part of the comprehensive management toolbox for monitoring water quality changes.

The 2016 HABHRCA report identifies monitoring challenges that include sustaining monitoring programs and maintaining the consistency of methods across monitoring programs. This study provides an opportunity for water quality managers and stakeholders to have a uniform satellite dataset (Schaeffer et al. 2015) and a consistent approach for determining lake, reservoir, and estuarine temperatures. Previous studies clearly demonstrate the connection between HAB responses, such as cyanobacteria growth (Robarts et al. 1987), toxin production (Davis et al. 2009), and dominance (Paerl et al. 2008; Paerl et al. 2013) in waters above 25°C. Therefore, the ability to provide synoptic spatial and temporal satellite measurements of temperature will remain a critical physical measurement in future efforts to forecast and monitor HABs across the nation. The Landsat SWT retrievals may be used with other variables to improve our understanding of HABs such as cyanobacterial blooms, overall water dynamics, and associated biochemical processes.

Global collections of lake, reservoir, and estuary SWT data may be possible in the near future as the NARR data were used for processing over North America, and global processing would include the Modern-Era Retrospective analysis for Research and Applications, Version 2 (MERRA-2) aerosol data for atmospheric characterization in the radiative transfer model. The results shown here demonstrated that new SWT data from satellites can be broadly applied to a vast number of lakes for monitoring and assessing changes throughout the decades.

## Figures and Tables

**Table 1.** Locations used for Landsat SWT validation.

Region	State	Waterbody	No. of stations

Coastal Plains	Florida	Lake Washington	3
	Florida	Lake Winder	2
	Texas	Lake Houston	3
Gulf of Mexico	Alabama	Mobile Bay	3
Mid-Atlantic	New Jersey	Barnegat Bay	7
	New Jersey	Coastal Ocean	6
	New Jersey	Manasquan Estuary	3
	New Jersey	Navesink River	5
	New Jersey	Newark Bay	6
	New Jersey	Raritan Bay	6
	New Jersey	Shark River	3
	New York	Hudson River	3
	New York	Jamaica Bay	9
Northern Appalachians	New York/Vermont	Lake Champlain	2
Northern Plains	North Dakota	Lake Audubon	3
	North Dakota	Lake Darling	1
Pacific	Washington	Puget Sound	3
South Atlantic	South Carolina	Cape Romaine Harbor	4
	South Carolina	Charleston Harbor	7
	South Carolina	Copahee Sound	2
	South Carolina	Murrells Inlet	2
	South Carolina	North Edisto River	5
	South Carolina	Port Royal Sound	2
	South Carolina	Santee Bay	2
	South Carolina	Sewee Bay	2
	South Carolina	St. Helena Sound	2
	South Carolina	Winyah Bay	1
Southern Appalachians	Arkansas	Lake Maumelle	2
	Arkansas	Lake Winona	1
	Virginia	Breckenridge Reservoir	3
Southern Plains	Kansas	Cheney Reservoir	1
	Oklahoma	Lake Texoma	4
	Texas	Aquilla Lake	3
	Texas	Lake Lyndon B. Johnson	2
	Texas	Lake Waco	3
Temperate Plains	Iowa	Arrow Head Lake	4
	Kansas	Clinton Lake	1
	Kansas	Perry Lake	1
Upper Midwest	Michigan	Cedar Hedge Lake	1
	Michigan	Duck Lake	2
	Michigan	Platte Lake	2
	Wisconsin	Big Crooked Lake	1
	Wisconsin	Crooked Lake	1
	Wisconsin	Green Lake	2
Western Mountains	California/Nevada	Lake Tahoe	8
	Colorado	Grand Lake	2
	Colorado	Lake Granby	3
	Colorado	Willow Creek Reservoir	1
	Oregon	Klamath Lake	2
	Washington	Bayley Lake	1
	Washington	Eunice Lake	1
	Washington	Lake Tapps	1
Xeric	California	Santa Rosa Creek Reservoir	1
	California	Sweetwater Reservoir	1
	Nevada	Walker Lake	6
	Nevada	Weber Reservoir	1
